# Noise exposure, hearing loss and cognitive impairment: a cross-sectional study based on an occupational health surveillance cohort in China

**DOI:** 10.3389/fpubh.2025.1455340

**Published:** 2025-02-19

**Authors:** Lei Huang, Linjuan Li, Juan Wang, Shushan Zhang, Huiyi Wu, Yajia Lan, Junying Li, Yang Zhang

**Affiliations:** ^1^Department of Postgraduate Students, West China Hospital and West China School of Medicine, Sichuan University, Chengdu, China; ^2^Thoracic Oncology Ward, Cancer Center, West China Hospital, Sichuan University/West China School of Nursing, Sichuan University, Chengdu, China; ^3^Occupational Health Examination Center, West China School of Public Health and West China Fourth Hospital, Sichuan University, Chengdu, China; ^4^Department of Neurology, Affiliated Hospital of North Sichuan Medical College, Nanchong, China; ^5^Department of Epidemiology and Biostatistics, West China School of Public Health and West China Fourth Hospital, Sichuan University, Chengdu, China; ^6^Department of Environmental Health and Occupational Medicine, West China School of Public Health and West China Fourth Hospital, Sichuan University, Chengdu, China; ^7^Department of Periodical Press and National Clinical Research Center for Geriatrics, West China Hospital, Sichuan University, Chengdu, China; ^8^Chinese Evidence-Based Medicine Center, West China Hospital, Sichuan University, Chengdu, China

**Keywords:** occupational exposure, noise dose, hearing loss, cognitive impairment, Alzheimer’s disease, structural equation modeling

## Abstract

**Background:**

High-intensity noise is associated with noise-induced hearing loss (NIHL). There is also evidence that noise exposure is related to cognitive impairment. This research aimed to analyze the associations and potential pathways of cumulative noise exposure (CNE), hearing loss and cognitive impairment.

**Methods:**

A total of 560 research subjects were included in this research from May 2021 to April 2022 in western China. The demographic features, occupational features, and CNE were investigated and examined. Hearing loss was evaluated according to the National standard GB/T 7583–1987 of China. The Mini-Mental State Examination (MMSE) and Montreal Cognitive Assessment (MoCA) were used to assess cognitive function. Structural equation modeling (SEM) was used to analyze the potential pathways and specific effect sizes of CNE, hearing loss and cognitive impairment.

**Results:**

The mean age of the research subjects was 34.3 (SD, 9.89). Men accounted for 96.4% (540/560) and women accounted for 3.6% (20/560). A total of 62.3% (349/560) held a college degree or above. The regression analysis showed that high dose CNE was related to MMSE (*β* = −1.069 (−1.539, −0.600)) and MoCA (*β* = −1.040 (−1.726, −0.355)) scores. The monaural threshold weighted value of the right ear (MTWV_R_) greater than 40 dB was associated with both MMSE (*β* = −1.183 (−2.033, −0.333)) and MoCA (*β* = −1.420 (−2.647, −0.193)) scores. The monaural threshold weighted value of the left ear (MTWV_L_) greater than 40 dB was also associated with MMSE (*β* = −1.540 (−2.389, −0.690)) and MoCA (*β* = −1.685 (−2.915, −0.456)) scores. The SEM result (Model C) showed that the standard effect of CNE- hearing loss path, CNE-MMSE path, and hearing loss-MMSE path were 0.142 (*p* < 0.001), −0.151 (*p* < 0.001), and −0.030 (*p* = 0.376). The Model D showed that the standard effect of CNE- hearing loss path, CNE- MoCA path, and hearing loss- MoCA path were 0.143 (*p* < 0.001), −0.048 (*p* = 0.267), and − 0.050 (*p* = 0.047). The CNE had only a direct effect on the MMSE score. Conversely, the CNE had only an indirect effect on the MoCA score, while hearing loss was borderline associated with MoCA. The total effects of CNE on MMSE and MoCA scores were −0.151 and −0.007, respectively.

**Conclusion:**

Job-related noise exposure is not only associated with NIHL but also with early cognitive impairment in occupational groups. However, there is not enough evidence indicating that NLHL mediates the associations.

## 1 Introduction

Noise is one of the most common occupational hazards. As an external stressor, it has been associated with multifaceted damage beyond the auditory system, such as sleep disorders (1) and cardiovascular disorders (2, 3). Epidemiological evidences also indicate a relationship between noise with different acoustic characteristics and negative impacts on nervous system such as annoyance and cognitive performance (4, 5). Exposure to noise peaks was associated with an increase in task error and longer reaction time, and subjects experienced greater discomfort, stress, and annoyance (6, 7). Irgens-Hansen et al. also reported that the reaction time of the noise group was significantly greater than that of the control group (8). Although community noise usually does not cause damage to the auditory system, it also have a negative impact on the nervous system and cognitive performance (9, 10). However, there is currently no agreement regarding the specific mechanism through which different types of noise exposure cause cognitive degradation. Several proposed mechanisms for noise-induced cognitive degradation include environment-genetic interactions, psychological stress, neuroexcitotoxicity, alterations in the microbiota-gut-brain axis, and neuroinflammation (11).

Hearing loss is linked to cognitive degradation. Clinical studies have confirmed that age-related hearing loss (ARHL) has been associated with cognitive degradation and Alzheimer’s disease (AD) in older adults (12, 13). A 17-year cohort study by Ford et al. revealed that after adjusting for other factors, the risk of dementia in individuals with hearing loss increased by 69% compared to that with normal hearing (14). Regarding the mechanism of hearing loss and dementia, it has been suggested that hearing loss can cause a series of changes in auditory system, including increased spontaneous activity and a wider frequency tuning range (15, 16), downregulation of synaptic inhibition and increased burst firing activity (17). Studies also suggested that hippocampal neurons can directly respond to these auditory stimulation signals (18, 19). In addition, NIHL can also cause a series of changes in both auditory cortex and hippocampus, including oxidative stress (20), neurotoxicity (21), and neuroinflammation (22), which may further lead to tau hyperphosphorylation and apoptosis in the hippocampus. Furthermore, communication disorders secondary to hearing loss can cause loneliness and social isolation, which further lead to cognitive degradation and dementia (23, 24). Cardin et al. suggested that the lack of cognitive resources caused by hearing loss is also a possible reason (25). Hearing loss arises from damage to the auditory system, and studies have pinpointed that aging, genetic mutations, noise exposure, ototoxic substances, and chronic diseases as significant risk factors (26). Long-term exposure to high-intensity noise can cause noise-induced hearing loss (NIHL) (27). Although both are hearing loss, NIHL is different from ARHL. ARHL is a type of sensor neural hearing loss caused by aging and degradation of the hearing system and is the most common sensory deficit problem in older adult individuals (28). However, NIHL is usually related to noise exposure, and the hearing threshold in the high-frequency band (4,000 Hz and 6,000 Hz) appears first and is the most severe. With the progression of NIHL, the speech frequency band is also damaged but is always less than that in the high-frequency band (29).

Few studies have focused on the risk and mechanism of NIHL and cognitive impairment. Only some experimental studies focusing on hearing loss and cognitive impairment have used high-intensity noise to establish animal models (30, 31). However, the essence of the abovementioned studies is still the relationship between noise and cognitive degradation. It cannot answer whether is the indirect effect of NIHL or the direct effect of noise (and/or noise-induced psychological stress, neuroinflammation, etc.). Whether the effects of NIHL and ARHL are consistent also cannot be conclusively determined. This involves the issue of hearing loss pathway and non-hearing loss pathway of noise. From the perspective of epidemiological evidence, even under lower-intensity noise conditions, such as community noise and road traffic noise, changes in the memory, reaction ability, and mood of different types of research subjects were still observed. Although the noise intensity is not high enough to cause NIHL, it can still affect the function of the nervous system, suggesting that non-hearing loss pathways may exist.

Is there a hearing loss pathway that leads to cognitive impairment under high-intensity noise exposure conditions? There are few studies on this topic. High-intensity noise exposure is common in occupational environments, especially in the manufacturing industry (32). However, studies on noise exposure and the risk of cognitive impairment have rarely focused on occupational exposure. Several studies have reported the relationship between job-related noise and cognitive impairment, but none of them monitored hearing loss (33, 34). Therefore, at the epidemiological level, current evidence cannot confirm whether there is a hearing loss pathway in the etiological chain of noise-induced cognitive degradation. Based on the aforementioned evidence, we pose the following questions pertaining to this research. Whether noise-induced hearing loss and noise-induced cognitive impairment belong to the same effect chain? Furthermore, whether NIHL play a mediating role in the etiological chain of noise and cognitive impairment? This research was conducted based on the above questions.

## 2 Methods

### 2.1 Research subjects

The research subjects were selected from the occupational health surveillance cohort of a large machinery and equipment manufacturing enterprise in western China. Prior to the study, sample size was estimated based on the expected level and standard deviation of cognitive function, the level of testing, and the efficacy of calibration. The power twomeans command was used to preliminarily estimate the sample size: the expected difference of cognitive function scores between groups was *Δ* = 0.5, the mean standard deviations were *σ* = 2.0, alpha = 0.05, and power = 0.80. The final estimate of the minimum sample size was 506. According to the estimated minimum sample size and the number of people in different department settings, a stratified random sampling method was adopted to select research subjects. The occupational group was divided into four departments, including Administration Department, Auxiliary Materials Department, General Assembly Department 1 and General Assembly Department 2. 710 research subjects were randomly selected by job ID according to the proportion of about 10%. This research was conducted from May 2021 to April 2022.

The inclusion criteria were as follows: (1) workers in the front-line and auxiliary production departments; (2) aged over 18 years, with working experience of no less than 6 months; (3) no serious trauma, physical disability, or sensory defect; (4) smooth communication and no language barriers; and (5) voluntary participation in this research under the premise of being fully informed. The exclusion criteria included the following: (1) a history of exposure to heavy metal elements such as manganese, lead, and copper; (2) previous or current use of drugs that may cause temporary or permanent neuropsychiatric symptoms; (3) secondary neuropsychiatric symptoms caused by trauma or surgery; (4) schizophrenia, drug abuse, or mental retardation; (5) a history of encephalitis or meningitis; and (6) suffering from other neurological diseases that may lead to cognitive degradation, such as epilepsy, cerebral infarction, or stroke.

**Figure 1:**
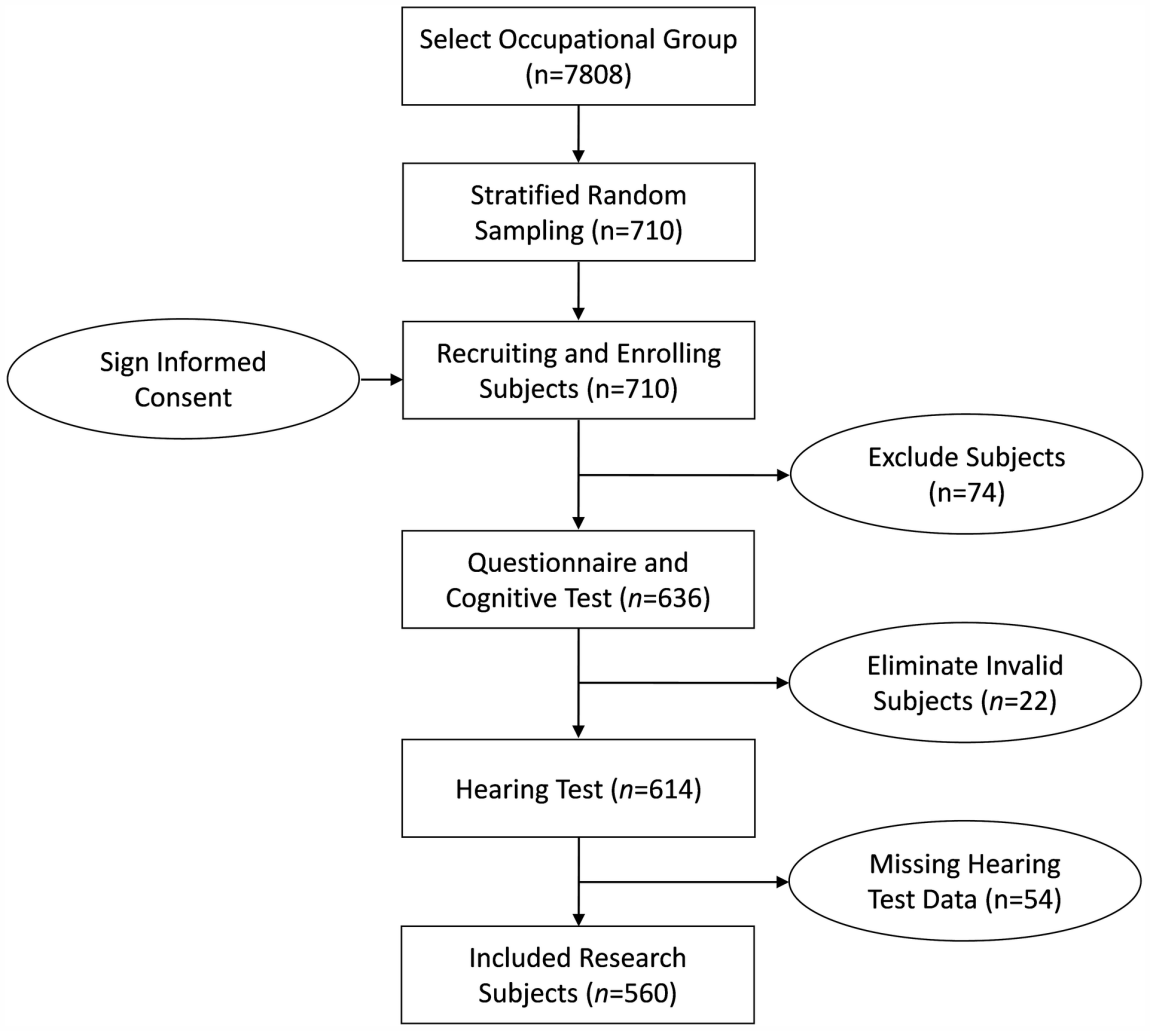
Flow diagram of the research subjects. 74 subjects did not meet the inclusion criteria (36 subjects have less than 6 months of work experience; 38 subjects disagreed to participate in this research). 22 subjects failed to complete all cognitive function scale tests (withdrew from cognitive tests). 54 subjects provided missing or incomplete hearing test results.

According to the inclusion and exclusion criteria, 74 were excluded. During the implementation of the study, 22 subjects failed to complete all cognitive function scale tests (withdrew from cognitive tests), and 54 subjects provided missing or incomplete hearing test results. After excluding invalid research subjects, a total of 560 research subjects met the inclusion criteria. The flow diagram of the research subjects is shown in Figure 1.

### 2.2 Demographic and occupational features

A self-designed questionnaire was administered to collect the demographic and occupational features of the research subjects. The demographic data included age, sex, marital status, education, living status, and monthly income. Occupational features include position change history, job descriptions, and employment duration. The investigators instructed the research subjects to complete a self-administered questionnaire.

### 2.3 Measurement of equivalent noise pressure level

A handheld sound level meter (class 1) and a personal noise dosimeter (class 2) were used for noise measurements. All the sound level meters adopted an A-weighting network, a 3 dB exchange rate, and a slow gear (SLOW). The spectrum used 1/1 octave, the filter was set to the Z level, and the detector was set to “linear.” The sound level meter was calibrated using a sound calibrator (114 dB, 1,000 Hz). Noise measurements and calculations were carried out according to the National Standard of China. Positions with complex noise conditions were measured using individual sampling, and the sampling period covered the entire working day as much as possible. Fixed-point sampling was used as an auxiliary measurement method for positions in a simple noise environment.

### 2.4 Calculation of cumulative noise exposure

According to the equal-energy principle of noise, the cumulative noise exposure (CNE) of different objects was calculated based on the noise intensity at different positions, with the working duration (cumulative days) as the weight. Owing to the position change and internal job transfer of some objects, detailed career history, including positions, job descriptions, and employment duration, was collected during the questionnaire survey. Those with incomplete or missing information were confirmed through face-to-face interviews or telephone visits. CNE was estimated using the following formula:


$$CNE=10\mathrm{lg}\left(\overset{n}{\underset{i=1}{\sum }}T_{i}10^{0.1{\left(L_{\mathrm{EX},8h}\right)}_{i}}\right)$$

*Note: CNE, cumulative noise exposure (dB.time).* ${L_{\mathrm{EX},8h}}_{i}$ *, noise equivalent sound level of job i in 8 h working day (dB(A)).* $T_{i}$ *, employment duration of job i (days). n, the total number of jobs.*

### 2.5 Audiometry and hearing threshold calculations

Audiometry was conducted according to the standard of the Acoustic Pure Tone Air Conduction Hearing Threshold Measurement for Hearing Protection (GB/T 7583-1987, China). The hearing thresholds of the participants were measured in a soundproof room. Pure-tone air conduction hearing threshold tests were conducted in a total of 6 frequency bands, including 500 Hz, 1,000 Hz, 2,000 Hz, 3,000 Hz, 4,000 Hz and 6,000 Hz. The left and right ears were tested separately. For research subjects with an abnormal pure tone air conduction hearing threshold test, a pure tone bone conduction hearing threshold test will be further performed to determine whether this is conductive hearing loss. In addition to disease history, interference from other non-noise factors, such as congenital atresia of the external auditory canal, cerumen, inflammation, tumors, drug-induced deafness, tympanic membrane rupture or perforation, middle ear deformity or inflammation, was excluded. According to the standard of Diagnosis of Occupational Noise Deafness (GBZ 49–2014, China), the binaural high frequency threshold average (HFAHT), monaural threshold weighted value of right ear (MTWV_R_), and monaural threshold weighted value of left ear (MTWV_L_) were calculated as follows, and divided into three levels: normal (<26 dB), mildly impaired (26 ~ dB), moderate or above impaired (40 ~ dB).


$$\mathrm{HFAHT}=\frac{\begin{array}{l} {HL}_{L3KHz}+{HL}_{L4KHz}+{HL}_{L6KHz}+{HL}_{R3KHz}\\ +{HL}_{R4KHz}+{HL}_{R6KHz} \end{array}}{6}$$

$$\mathrm{MTWV}=\left(\frac{{HL}_{500\mathrm{Hz}}+{HL}_{1KHz}+{HL}_{2KHz}}{3}\right)\times 0.9+{HL}_{4KHz}\times 0.1$$

*Note: HFAHT, binaural high frequency threshold average. MTWV, monaural threshold weighted value.* HL *, hearing level (dB).* $HL_{L}$ *, hearing level of the left ear (dB).* $HL_{R}$ *, hearing level of the right ear (dB).*

### 2.6 Cognitive function test

The Chinese version of the Mini-Mental State Examination Scale (MMSE) (35) and Montreal Cognitive Assessment Scale (MoCA) (36) were used to assess the cognitive function of the subjects. The MMSE was used to assess the orientation, memory, attention, calculation, and language skills of the participants (37). The MoCA was used to assess attention, executive function, memory, language, visual structure, abstract thinking, and orientation (38). The total scores of the MMSE and MoCA are 30 points. The raw MMSE and MoCA scores were used for statistical analysis in this research.

### 2.7 Statistical analysis

STATA 14.0 software was used for statistical analysis. The Kolmogorov–Smirnov normality test was used to assess the distribution of cognitive function, the test level was *α* = 0.05. The Mann–Whitney test (two groups) and Kruskal–Wallis test (multiple groups) were used to compare the cognitive function levels of different demographic subgroups, CNE subgroups and hearing threshold groups. Demographic factors, CNE and hearing loss were analyzed as categorical, MoCA and MMSE were analyzed as numerical in all the analysis. Single factor regression model and baseline adjusted regression model was used to analysis the relationship between CNE, hearing loss and cognitive function. The baseline model adjusted for age, sex, education, marital status, living status, and monthly income. Structural equation modeling (SEM) was used to analyze the potential pathways and specific effect sizes of CNE, hearing loss and cognitive impairment. The SEM can also be used to identify intermediate factors in a specific model. Hearing loss was set as a latent variable, which consisted of HFAHT, MTWVR, and MTWVL. The full models adjusted for age, sex, education, marital status, living status, and monthly income. The fitting parameters for SEM include the ratio of chi-square degrees of freedom (χ^2^/df, <2), goodness-of-fit index (GFI, >0.9), normed fit index (NFI, >0.9), relative fit index (RFI, >0.9), comparative fit index (CFI, >0.9), incremental fit index (IFI, >0.9) and root mean square error of approximation (RMSEA, <0.05). The models need repeated fitting and correction to check whether the fitting parameters meet the standards. If the fitting parameters met the above criteria, the regression model was considered to be an ideal model.

## 3 Results

### 3.1 Basic characteristics of the research subjects

A total of 560 research subjects were enrolled in the study, ranging in age from 19 to 59 years, with a mean age of 34.3 (SD, 9.89). Men accounted for 96.4% (540/560) of the participants, and women accounted for 3.6% (20/560) of the participants. In terms of marital status, 36.4% (204/560) were unmarried or single subjects, and 63.6% (356/560) were married. A total of 62.3% (349/560) had a college degree or above. Approximately 16.1% (90/560) of the research subjects chose to live alone, while 83.9% (470/560) chose to live with their family. A total of 87.4% (489/560) of the participants’ incomes were between ¥ 2,500 and 8,000. A total of 59.5% (333/560) of the subjects were in the medium noise dose group, and 25.4% (142/560) were in the high noise dose group. A total of 17.9% (100/560), 2.5% (14/560) and 2.7% (15/560) of the research subjects had HFAHT, MTWV_R_, and MTWV_L_ greater than 40 dB, respectively (Table 1).

**Table 1:** Cognitive function in different subgroups.

Variable	N (%)	MMSE			W/H	*p* value	MoCA			W/H	*p* value
		Mean	Median	IQR			Mean	Median	IQR		
**Age (year)**					**75.47**	**<0.001**				**104.80**	**<0.001**
<25	101 (18.0)	28.8 (1.13)	29	2			26.7 (1.77)	27	2		
25~	226 (40.4)	28.7 (1.30)	29	2			26.3 (2.06)	26	3		
35~	111 (19.8)	28.0 (1.90)	28	2			24.9 (2.50)	25	4		
45~	122 (21.8)	27.2 (1.99)	27	3			23.7 (2.93)	24	4		
**Sex**					0.31	0.540				0.46	0.470
Male	540 (96.4)	28.2 (1.70)	29	1.5			25.5 (2.58)	26	3		
Female	20 (3.6)	28.2 (1.69)	28.5	1.5			25.8 (2.61)	26	2.5		
**Education**					**34.81**	**<0.001**				**92.08**	**<0.001**
Junior high school or below	37 (6.6)	27.2 (1.96)	27	3			22.9 (2.84)	23	3		
High school	174 (31.1)	27.9 (1.72)	28	2			24.6 (2.42)	25	3		
Bachelor degree or above	349 (62.3)	28.6 (1.54)	29	2			26.3 (2.27)	26	3		
**Marital status**					**4.80**	**<0.001**				**6.60**	**<0.001**
Unmarried or single	204 (36.4)	28.7 (1.37)	29	2			26.5 (2.01)	27	3		
Married	356 (63.6)	28.0 (1.80)	28	2			25.0 (2.70)	25	3		
**Living status**					0.08	0.958				0.27	0.743
Live alone	90 (16.1)	28.2 (1.93)	29	2			25.4 (3.00)	26	3		
Live with family	470 (83.9)	28.3 (1.62)	29	3			25.6 (2.48)	26	3		
**Monthly income (¥)**					3.42	0.438				**15.06**	**0.004**
<2,500	26 (4.6)	28.0 (1.56)	28	2			24.9 (2.45)	26	4		
2,500~	239 (42.7)	28.2 (1.75)	29	2			25.1 (2.52)	25.5	3		
5,000~	250 (44.6)	28.3 (1.70)	29	3			25.9 (2.68)	26	4		
8,000~	31 (5.5)	28.7 (1.24)	29	2			26.1 (1.71)	26	2		
10,000~	14 (2.5)	28.4 (1.22)	28.5	1			25.9 (2.03)	26	2		
**CNE (dB.time)**					**44.57**	**<0.001**				**19.60**	**<0.001**
Low dose (<107.8)	85 (15.2)	28.8 (1.15)	29	2			26.2 (1.74)	26	2		
Medium dose (107.8~)	333 (59.5)	28.4 (1.60)	29	2			25.7 (2.57)	26	4		
High dose (127.8~)	142 (25.4)	27.5 (1.97)	28	2			24.7 (2.85)	25	4		
**HFAHT (dB)**					**36.26**	**<0.001**				**51.46**	**<0.001**
<26	365 (65.1)	28.5 (1.50)	29	2			26.1 (2.22)	26	3		
26~	95 (17.0)	28.2 (1.74)	29	3			24.9 (2.70)	25	3		
40~	100 (17.9)	27.4 (2.03)	28	2			24.0 (2.97)	24	4		
**MTWV**_**R**_ **(dB)**					**23.75**	**<0.001**				**28.24**	**<0.001**
<26	467 (83.4)	28.4 (1.54)	29	2			25.8 (2.40)	26	4		
26~	79 (14.1)	27.6 (2.10)	28	2			24.4 (2.84)	25	3		
40~	14 (2.5)	26.5 (2.28)	27	3			22.9 (3.69)	24	4		
**MTWV**_**L**_ **(dB)**					**32.08**	**<0.001**				**39.01**	**<0.001**
<26	460 (82.1)	28.4 (1.54)	29	2			25.8 (2.37)	26	3		
26~	85 (15.2)	27.6 (1.79)	28	2			24.2 (2.83)	24	4		
40~	15 (2.7)	25.9 (2.94)	27	4			22.7 (3.51)	24	5		

MMSE, Mini-Mental State Examination; MoCA, Montreal Cognitive Assessment; CNE, cumulative noise exposure; HFAHT, binaural high-frequency threshold average; MTWV_R_, monaural threshold weighted value of the right ear; MTWV_L_, monaural threshold weighted value of the left ear; dB, decibel; IQR, Interquartile Range; W/H, The statistic of Mann–Whitney test (two groups) and Kruskal–Wallis test (multiple groups).

The Kolmogorov–Smirnov normality test showed that the MMSE and MoCA scores did not conform to the normal distribution (*p* < 0.05). The results of the Kruskal–Wallis test and Mann–Whitney test showed that there were differences in both MMSE and MoCA scores among the different age, education, and marital status subgroups (*p* < 0.05). Age was negatively correlated with cognitive function, and education was positively correlated with cognitive function. There was a significant difference in the MoCA score between the monthly income subgroups (*p* < 0.05). No significant difference was found in either MMSE or MoCA scores between the sex and living status subgroups. There were significant differences in both MMSE and MoCA scores among the different CNE, HFAHT, MTWV_R_, and MTWV_L_ subgroups (*p* < 0.05) (Table 1). CNE and hearing loss were negatively correlated with cognitive function.

### 3.2 Regression analysis of noise exposure, hearing loss and cognitive function

As evident from Table 2, CNE was associated with both hearing loss (HFAHT, MTWV_R_, and MTWV_L_) and cognitive function (*p* < 0.05, Table 2). The baseline adjusted single-factor regression model showed that the CNE high-dose group had statistical significance in the regression models of the MMSE and MoCA scores. Compared with those of the CNE low-dose group, the MMSE and MoCA scores of the high-dose group were reduced by 1.069 (−1.539, −0.600) and 1.040 (−1.726, −0.355) points, respectively (Table 3). The MTWV_R_ greater than 40 dB was associated with both MMSE (*β* = −1.183 (−2.033, −0.333), *p* = 0.006) and MoCA (*β* = −1.420 (−2.647, −0.193), *p* = 0.023) scores. The MTWV_L_ greater than 40 dB was associated with both MMSE (*β* = −1.540 (−2.389, −0.690), *p* < 0.001) and MoCA (*β* = −1.685 (−2.915, −0.456), *p* = 0.007) scores. The HFAHT had no statistical significance in the regression models of either MMSE or MoCA scores.

**Table 2:** Cognitive function in different subgroups.

Variable	N (%)	HFAHT (dB)			MTWV_R_ (dB)			MTWV_L_ (dB)			MMSE			MoCA		
		Mean	Median	IQR	Mean	Median	IQR	Mean	Median	IQR	Mean	Median	IQR	Mean	Median	IQR
CNE (dB.time)																
Low dose (<107.8)	85 (15.2)	23.7 (10.27)	20	4	19.5 (4.32)	20	4.5	19.9 (4.47)	20.5	4.5	28.8 (1.15)	29	2	26.2 (1.74)	26	2
Medium dose (107.8~)	333 (59.5)	26.6 (12.04)	23	9	20.8 (6.18)	20	5	21.3 (5.72)	20.5	3.5	28.4 (1.60)	29	2	25.7 (2.57)	26	4
High dose (127.8~)	142 (25.4)	36.6 (20.27)	28	29	24.4 (10.82)	22	7	24.7 (9.51)	22	7	27.5 (1.97)	28	2	24.7 (2.85)	25	4
**H**		**39.72**			**20.16**			**34.18**			**44.57**			**19.60**		
***p* value**		**<0.001**			**<0.001**			**<0.001**			**<0.001**			**<0.001**		

MMSE, Mini-Mental State Examination; MoCA, Montreal Cognitive Assessment; CNE, cumulative noise exposure; HFAHT, binaural high-frequency threshold average; MTWV_R_, monaural threshold weighted value of the right ear; MTWV_L_, monaural threshold weighted value of the left ear; dB, decibel; IQR, Interquartile Range; H, The statistic of Kruskal–Wallis test.

**Table 3:** Regression analysis of noise exposure, hearing loss and cognitive function.

Variable	MMSE			MoCA		
	B	95%CI	*p* value	B	95%CI	*p* value
Single factor regression model						
CNE (dB.time)						
Low dose	Ref			Ref		
Medium dose	−0.311	(−0.701, 0.078)	0.118	−0.471	(−1.076, 0.134)	0.127
High dose	−1.288	(−1.728, −0.847)	**<0.001**	−1.460	(−2.143, −0.777)	**<0.001**
HFAHT (dB)						
<26	Ref			Ref		
26~	−0.333	(−0.705, 0.039)	0.079	−1.129	(−1.684, −0.575)	**<0.001**
40~	−1.141	(−1.506, −0.777)	**<0.001**	−2.076	(−2.620, −1.533)	**<0.001**
MTWV_R_ (dB)						
<26	Ref			Ref		
26~	−0.793	(−1.188, −0.398)	**<0.001**	−1.404	(−2.002, −0.805)	**<0.001**
40~	−1.900	(−2.782, −1.020)	**<0.001**	−2.855	(−4.189, −1.521)	**<0.001**
MTWV_L_ (dB)						
<26	Ref			Ref		
26~	−0.779	(−1.158, −0.400)	**<0.001**	−1.606	(−2.181, −1.031)	**<0.001**
40~	−2.493	(−3.334, −1.651)	**<0.001**	−3.108	(−4.385, −1.831)	**<0.001**
Baseline adjusted single-factor regression model						
CNE (dB.time)						
Low dose	Ref			Ref		
Medium dose	−0.205	(−0.575, 0.165)	0.277	−0.261	(−0.801, 0.279)	0.343
High dose	−1.069	(−1.539, −0.600)	**<0.001**	−1.040	(−1.726, −0.355)	**0.003**
HFAHT (dB)						
<26	Ref			Ref		
26~	0.268	(−0.120, 0.657)	0.175	−0.119	(−0.680, 0.442)	0.677
40~	−0.197	(−0.630, 0.237)	0.373	−0.375	(−1.000, 0.251)	0.240
MTWV_R_ (dB)						
<26	Ref			Ref		
26~	0.032	(−0.398, 0.462)	0.885	0.081	(−0.539, 0.702)	0.797
40~	−1.183	(−2.033, −0.333)	**0.006**	−1.420	(−2.647, −0.193)	**0.023**
MTWV_L_ (dB)						
<26	Ref			Ref		
26~	−0.009	(−0.432, 0.413)	0.966	−0.007	(−0.619, 0.604)	0.981
40~	−1.540	(−2.389, −0.690)	**<0.001**	−1.685	(−2.915, −0.456)	**0.007**

The baseline adjusted single-factor regression model was used in this part. Each model was adjusted for age, sex, education, marital status, living status, and monthly income. MMSE, Mini-Mental State Examination; MoCA, Montreal Cognitive Assessment; CNE, cumulative noise exposure; HFAHT, binaural high-frequency threshold average; MTWV_R_, monaural threshold weighted value of the right ear; MTWV_L_, monaural threshold weighted value of the left ear; dB, decibel.

### 3.3 Structural equation modeling of CNE, hearing loss and cognitive function

To explore the relationships between noise exposure, hearing loss and cognitive function and their potential pathways, this research used SEM to conduct path analyses. In the basic models (Models A and B), CNE was set as an exogenous variable, and hearing loss was set as a latent variable, which consisted of HFAHT, MTWV_R_, and MTWV_L_. MMSE and MoCA scores were used as the dependent variables of the SEMs. The final fitting models (Table 4) met the criteria after repeated fittings and corrections (χ^2^/df < 2, RMSEA <0.05, GFI > 0.9, NFI > 0.9, RFI > 0.9, CFI > 0.95, and IFI > 0.9).

**Table 4:** Basic models of CNE, hearing loss and cognitive function.

Models and paths	Effect	Standard effect	*p* value
Model A: CNE-hearing loss-MMSE			
CNE-hearing loss	0.393	0.246	**<0.001**
CNE-MMSE	−0.025	−0.161	**<0.001**
Hearing loss-MMSE	−0.020	−0.202	**<0.001**
Model B: CNE-hearing loss-MoCA			
CNE-hearing loss	0.395	0.249	**<0.001**
CNE-MoCA	−0.010	−0.041	0.327
Hearing loss-MoCA	−0.042	−0.281	**<0.001**

CNE, cumulative noise exposure; MMSE, Mini-Mental State Examination; MoCA, Montreal Cognitive Assessment.

**Figure 2:**
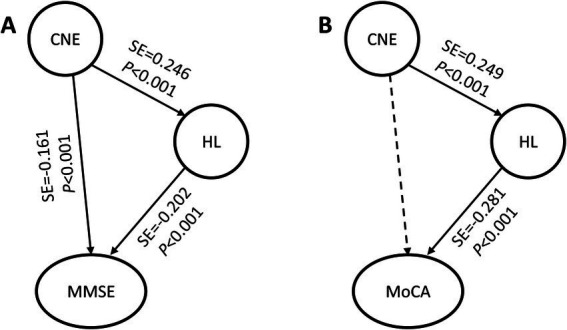
Basic models of CNE, hearing loss and cognitive function. **(A)** Basic model of CNE-hearing loss-MMSE; **(B)** Basic model of CNE-hearing loss-MoCA. CNE, cumulative noise exposure; MMSE, Mini-Mental State Examination; MoCA, Montreal Cognitive Assessment; HL, latent variable of hearing loss; SE, standard effect. The solid arrow indicates that the path was meaningful, and the dotted arrow indicates that the path was not statistically significant.

The SEM results of the MMSE (Table 4, Model A) showed that the CNE-hearing loss, CNE-MMSE and hearing loss-MMSE paths were all statistically significant (*p* < 0.05). The SEM results of the MoCA (Table 4, Model B) showed that the CNE-hearing loss and hearing loss-MoCA paths were statistically significant (*p* < 0.05, Figure 2A), but no statistical difference was found in the CNE-MoCA path (Figure 2B).

There is much evidence that demographic characteristics are associated with cognitive degradation, especially age-related factors. Therefore, the study further included demographic variables, including age, sex, education, marital status, living status, and monthly income, and used the SEM method to analyze the significant factors and their potential pathways. According to the SEM fitting standards, the full models (Table 5) incorporating demographic variables were fitted and modified to obtain the optimal models (χ^2^/df < 2, RMSEA <0.05, GFI > 0.9, NFI > 0.9, RFI > 0.9, CFI > 0.95, and IFI > 0.9).

**Table 5:** Full models of CNE, hearing loss and cognitive function.

Models and paths	Effect	Standard effect	*p* value
Model C: CNE-hearing loss-MMSE			
Age-CNE	0.439	0.381	**<0.001**
Sex-CNE	0.383	0.006	0.867
Education-CNE	5.042	0.272	**<0.001**
Age-hearing loss	0.765	0.418	**<0.001**
Sex-hearing loss	−6.420	−0.066	**0.017**
Education-hearing loss	−2.520	−0.086	**0.003**
CNE-hearing loss	0.226	0.142	**<0.001**
Age-MMSE	−0.046	−0.273	**<0.001**
Sex-MMSE	−0.097	−0.011	0.784
Education-MMSE	0.296	0.108	**0.008**
CNE-MMSE	−0.022	−0.151	**<0.001**
Hearing loss-MMSE	−0.003	−0.030	0.376
Model D: CNE-hearing loss-MoCA			
Age-CNE	0.439	0.381	**<0.001**
Sex-CNE	0.383	0.006	0.867
Education-CNE	5.042	0.272	**<0.001**
Age-hearing loss	0.766	0.420	**<0.001**
Sex-hearing loss	−6.438	−0.066	**0.017**
Education-hearing loss	−2.514	−0.086	**0.003**
CNE-hearing loss	0.226	0.143	**<0.001**
Age-MoCA	−0.078	−0.310	**<0.001**
Sex-MoCA	0.115	0.009	0.822
Education-MoCA	1.007	0.250	**<0.001**
CNE-MoCA	−0.010	−0.048	0.267
Hearing loss-MoCA	−0.007	−0.050	**0.047**

CNE, cumulative noise exposure; MMSE, Mini-Mental State Examination; MoCA, Montreal Cognitive Assessment.

The full models of the MMSE and MoCA (Table 5, Models C and D) showed that age-related paths and education-related paths were all statistically significant (*p* < 0.05), suggesting that age-related factors and education had significant impact on both the auditory system and cognitive function. The sex-hearing loss paths were statistically significant in both the MMSE and MoCA models (*p* < 0.05). The SEM results of the MMSE (Table 5, Model C) showed that the CNE-hearing loss and CNE-MMSE paths were both statistically significant (*p* < 0.05), while the hearing loss-MMSE path had no statistical significance. The CNE had a direct effect on only the MMSE (direct effect value was −0.151, Table 6). The SEM results of the MoCA (Table 5, Model D) showed that the CNE-hearing loss and hearing loss-MoCA paths were both statistically significant, but CNE was borderline associated with MoCA (*p* = 0.047). The CNE-MoCA path was not statistically significant, which was consistent with the results of the basic model (Table 4, Model B). The indirect effect of the CNE on the MoCA score was −0.007, while the direct effect of hearing loss on the MoCA score was −0.050 (Table 6). Judging from the total effect, age was still the most influential factor. The total effects of age on MMSE and MoCA scores were − 0.345 and − 0.352, respectively. The total effects of CNE on MMSE and MoCA scores were − 0.151 and − 0.007, respectively, and the effect of CNE on MMSE scores was greater than that on MoCA scores (Table 6).

**Table 6:** Effect of demographic characteristics, CNE and hearing loss on cognitive function.

Models and variables	Standard effect		
	Direct effect	Indirect effect	Total effect
Model C: CNE-hearing loss-MMSE			
Age	−0.273	−0.072	−0.345
Sex	0	0	0
Education	0.108	−0.041	0.067
CNE	−0.151	0	−0.151
Hearing loss	0	–	0
Model D: CNE-hearing loss-MoCA			
Age	−0.310	−0.042	−0.352
Sex	0	0.003	0.003
Education	0.250	−0.002	0.248
CNE	0	−0.007	−0.007
Hearing loss	−0.050	–	−0.050

CNE, cumulative noise exposure; MMSE, Mini-Mental State Examination; MoCA, Montreal Cognitive Assessment. Nonsignificant paths have been deleted, and the effect values corresponding to the paths are not included in the total effect.

Without setting up latent variables, the relationships and potential pathways between noise exposure, hearing loss (HFAHT, MTWV_R_ and MTWV_L_) and cognitive function were further analyzed using SEM (Supplementary Table S2). The SEM results showed that the effect of the HFAHT on the MoCA score was greater than the effect on the MMSE score, and the effect of the MTWV_L_ on cognitive function was greater than that of the MTWV_R_.

## 4 Discussion

This research discovered that CNE exhibited a negative correlation with both MMSE and MoCA scores, while hearing loss demonstrated a positive relationship with cognitive scores. The MTWV_L_ and MTWV_R_ greater than 40 dB were correlated with both MMSE and MoCA scores, while HFAHT did not demonstrate a statistically significant correlation. The SEM analysis revealed that CNE had a direct effect solely on the MMSE score. Conversely, CNE’s impact on the MoCA score was indirect, but there is not enough evidence indicating that HL mediates these associations. The total effects of CNE on MMSE and MoCA scores amounted to −0.151 and −0.007, respectively. Furthermore, the effect of MTWV_L_ on cognitive function was more pronounced than that of MTWV_R_.

Based on noise surveillance data and career history of the research subjects, this research quantitatively assessed job-related CNE to analyze the risk of hearing loss and cognitive impairment. Generally, an individual’s working life can last as long as 30 to 40 years. Job-related noise exposure is often long-term and cumulative, and the health damage caused by noise is also progressive. Cross-sectional exposure data cannot reflect cumulative exposure, and there may be bias in explaining the association between noise exposure and outcomes. However, the CNE can more accurately reveal the association between exposure and outcomes (39).

Previous studies have shown that noise exposure is associated with cognitive impairment (11). Clinical studies have also confirmed that ARHL is associated with cognitive impairment and dementia (40). Regression analysis in this research revealed that in addition to the CNE, the NIHL was also associated with cognitive impairment. Notably, we found that HFAHT did not appear to be associated with cognitive impairment, but both MTWV_R_ and MTWV_L_ were correlated with cognitive impairment. We reviewed evidence on asymmetric hearing loss (AHL) regarding discrepancies in the association between binaural ears and cognitive impairment. Studies have demonstrated that NIHL is often more severe in the left ear, which seems to be more susceptible to job-related noise compared to the right ear, despite the underlying causes of this phenomenon remaining unclear (41–43). In addition, there are different opinions. Aarhus et al. reported that there was no association between job-related noise exposure and AHL (44). Further research revealed that the reason for this difference may be related to the habitual position of the head during work. The fixed work position led to differences in the noise received by the left and right ears. Approximately 90% of people are right-handed (45), and right-handed people may be more likely to point their left ear toward the noise source during work (46). Most workers habitually turn to the right when operating noisy equipment, so the left ear is more susceptible to equipment noise (29, 47).

**Figure 3:**
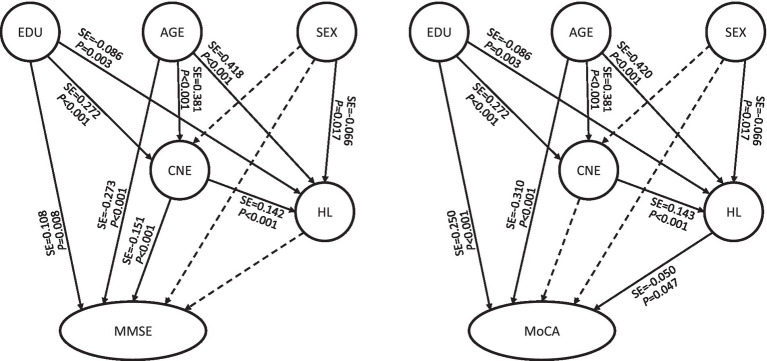
Full models of CNE, hearing loss and cognitive function. CNE, cumulative noise exposure; MMSE, Mini-Mental State Examination; MoCA, Montreal Cognitive Assessment; HL, latent variable of hearing loss; SE, standard effect; EDU, education. The solid arrow indicates that the path was meaningful, and the dotted arrow indicates that the path was not statistically significant.

Based on the above evidence, specifically the AHL caused by job-related noise exposure, coupled with the asymmetry observed in the association between binaural hearing loss and cognitive impairment in this research, it has been further demonstrated that job-related noise is related to cognitive impairment. Hearing loss may play an important role or potentially serve as an indirect risk factor. This research further employed SEM to investigate the associations between CNE, hearing loss and cognitive impairment, as well as the potential underlying pathways. The basic MMSE model showed that both the CNE-MMSE and the CNE-hearing loss-MMSE paths were statistically significant. The basic MoCA model showed that the CNE-hearing loss-MoCA path was statistically significant, but no statistically significant difference was found in the CNE-MoCA path. The full models showed that except for the CNE-hearing loss-MMSE path, which did not reach significance, the other paths were consistent with the basic models, but hearing loss was borderline associated with MoCA (Figure 3). The MMSE score seems to be insensitive to hearing loss and is only directly affected by the CNE, while the MoCA score tends to be affected by hearing loss. However, there is no enough evidence indicating that hearing loss mediates the associations.

The difference in SEM results between the MMSE and MoCA may be attributed to the different emphases of the MMSE and MoCA scales. Numerous studies have shown, that compared to the MMSE, the MoCA is a more sensitive tool for detecting neurocognitive disorders and is more suitable for detecting early impairment of cognitive function (48, 49). The MMSE focuses on the assessment of short-term memory and language functions while ignoring executive functions. People with a normal MMSE score may perform poorly on the MoCA (50). In contrast, the MoCA adds content such as logical connections, stereoscopic views, and clock drawing tests to evaluate executive functions and visuospatial abilities.

Based on the above evidences, it is speculated that the short-term memory and language functions that the MMSE focuses on may be more susceptible to short-term noise exposure and are not sensitive to hearing loss. The executive functions and visuospatial abilities that the MoCA focused on may be affected by long-term noise exposure. However, how hearing loss leads to changes in the nervous system, especially the hippocampus, is not yet fully understood. Relevant studies suggested that hearing loss could cause a series of secondary changes in the auditory system, which in turn can cause changes in cognitive function areas. Hippocampal neurons can even respond directly to auditory stimulation signals (18, 19), but the path by which auditory signals reach the hippocampus is not fully understood and requires further study.

Limitations: (1) Cognitive function data were only cross-sectional data based on the limited research duration. (2) The outcome variable was early impairment of cognitive function, and disease outcomes such as mild cognitive impairment and dementia could not be tracked during the research duration. (3) Selection bias may exist because quite a lot of subjects were excluded or did not participate in this research. (4) Since there are fewer female employees in the enterprise, we did not recruit more female subjects to further analyze the effect of gender. Research prospects: (1) Continue to conduct longitudinal follow-up studies to further investigate the impact of chronic noise exposure on the initiation and progression of dementia. (2) Further search for evidence on whether NIHL plays a mediating role in the chain of noise and cognitive impairment.

In summary, this study revealed that job-related noise exposure is not only associated with NIHL but also with early impairment of cognitive function in occupational groups. CNE had a direct effect on MMSE score and a marginal indirect effect on MoCA score. However, insufficient evidence suggests that NLHL mediates the associations. The findings of this research offer valuable insights for the long-term prevention and treatment of cognitive degradation and dementia. Nevertheless, further studies are required to elucidate the causal association and the mechanism of noise, NIHL and cognitive impairment. A key focus of the studies should be to delve deeper into the parameters of sound signals, such as peak levels, temporal variations, amplitude modulation, impulsivity, and frequency distribution, and to elucidate their impacts on cognitive function. Additionally, future research is needed to determine whether cognitive impairment closely associated with noise exposure increase the risk of dementia in later life.

## Data Availability

The original contributions presented in the study are included in the article/Supplementary material, further inquiries can be directed to the corresponding author.
