# Supplementary material for: Noise exposure, hearing loss and cognitive impairment: a cross-sectional study based on an occupational health surveillance cohort in China
Source: Front Public Health. 2025 Feb 19;13:1455340. doi: 10.3389/fpubh.2025.1455340 (PMC11880209; doi:10.3389/fpubh.2025.1455340)
Supplement: Supplementary file 1 [file Table_1.docx]

**Table S1** Regression analysis of noise exposure, hearing loss and cognitive function (sensitivity analysis)

| **Variable** | **MMSE** | | |  | **MoCA** | | |
| --- | --- | --- | --- | --- | --- | --- | --- |
|  | **B** | **95%CI** | ***p*** **Value** |  | **B** | **95%CI** | ***p*** **Value** |
| **CNE (dB.time)** |  |  |  |  |  |  |  |
| low dose | Ref |  |  |  | Ref |  |  |
| medium dose | -0.173 | (-0.493, 0.147) | 0.288 |  | -0.267 | (-0.732, 0.199) | 0.261 |
| high dose | -1.073 | (-1.492, -0.655) | **<0.001** |  | -1.127 | (-1.737, -0.518) | **<0.001** |
| **HFAHT (dB)** |  |  |  |  |  |  |  |
| <26 | Ref |  |  |  | Ref |  |  |
| 26~ | 0.309 | (-0.012, 0.629) | 0.059 |  | -0.071 | (-0.391, 0.533) | 0.763 |
| 40~ | -0.239 | (-0.658, 0.180) | 0.262 |  | -0.384 | (-0.987, 0.220) | 0.212 |
| **MTWV_R_ (dB)** |  |  |  |  |  |  |  |
| <26 | Ref |  |  |  | Ref |  |  |
| 26~ | 0.173 | (-0.155, 0.501) | 0.300 |  | 0.246 | (-0.225, 0.718) | 0.305 |
| 40~ | -1.171 | (-2.010, -0.332) | **0.006** |  | -1.420 | (-2.627, -0.212) | **0.021** |
| **MTWV_L_ (dB)** |  |  |  |  |  |  |  |
| <26 | Ref |  |  |  | Ref |  |  |
| 26~ | 0.143 | (-0.180, 0.468) | 0.385 |  | 0.186 | (-0.281, 0.655) | 0.434 |
| 40~ | -1.528 | (-2.366, -0.689) | **<0.001** |  | -1.677 | (-2.887, -0.466) | **0.007** |

*Note: The baseline adjusted single-factor regression model was used in this part. Each model was adjusted for age, sex, education, marital status, living status, and monthly income. MMSE, Mini-Mental State Examination. MoCA, Montreal Cognitive Assessment. CNE, cumulative noise exposure. HFAHT, binaural high-frequency threshold average. MTWV_R_, monaural threshold weighted value of the right ear. MTWV_L_, monaural threshold weighted value of the left ear.*

**Table S2** Full models of CNE, HFAHT, MTWV_R_, MTWV_L_ and cognitive function

| **Models and paths** | | **Effect** | **Standard effect** | ***p*** **Value** |
| --- | --- | --- | --- | --- |
| **Model E: CNE-HFAHT-MMSE** | | | | |
|  | CNE-HFAHT | 0.193 | 0.138 | **<0.001** |
|  | CNE-MMSE | -0.018 | -0.112 | **0.006** |
|  | HFAHT-MMSE | -0.007 | -0.06 | 0.224 |
| **Model F: CNE-HFAHT-MoCA** | | | | |
|  | CNE-HFAHT | 0.193 | 0.138 | **<0.001** |
|  | CNE-MoCA | 0.005 | 0.021 | 0.589 |
|  | HFAHT-MoCA | -0.017 | -0.103 | **0.031** |
| **Model G: CNE-MTWV_R_-MMSE** | | | | |
|  | CNE-MTWV_R_ | 0.069 | 0.098 | **0.012** |
|  | CNE-MMSE | -0.018 | -0.116 | **0.004** |
|  | MTWV_R_-MMSE | -0.01 | -0.043 | 0.324 |
| **Model H: CNE-MTWV_R_-MoCA** | | | | |
|  | CNE-MTWV_R_ | 0.069 | 0.098 | **0.012** |
|  | CNE-MoCA | 0.004 | 0.015 | 0.707 |
|  | MTWV_R_—MoCA | -0.026 | -0.077 | 0.068 |
| **Model I: CNE-MTWV_L_-MMSE** | | | | |
|  | CNE-MTWV_L_ | 0.076 | 0.119 | **0.002** |
|  | CNE-MMSE | -0.017 | -0.111 | **0.006** |
|  | MTWV_L_-MMSE | -0.02 | -0.080 | 0.069 |
| **Model J: CNE-MTWV_L_-MoCA** | | | | |
|  | CNE-MTWV_L_ | 0.076 | 0.119 | **0.002** |
|  | CNE-MoCA | 0.005 | 0.019 | 0.621 |
|  | MTWV_L_-MoCA | -0.038 | -0.103 | **0.015** |

*Note: CNE, cumulative noise exposure. MMSE, Mini-Mental State Examination. MoCA, Montreal Cognitive Assessment. HFAHT, binaural high-frequency threshold average. MTWV_R_, monaural threshold weighted value of the right ear. MTWV_L_, monaural threshold weighted value of the left ear. Pathways of demographic variables are not shown in the table.*
